# An unusual case of Waldenstrom’s macroglobulinemia presented with nasopharyngeal involvement

**DOI:** 10.3332/ecancer.2013.362

**Published:** 2013-10-16

**Authors:** Vishwanath Sathyanarayanan, Umesh Das, BS Shankaranand, Sumit Gupta, Naveen J Anvekar, KC Lakshmaiah

**Affiliations:** 1 Department of Medical Oncology, Kidwai Memorial Institute of Oncology, Bangalore, Karnataka, India; 2 Department of Pathology, Kidwai Memorial Institute of Oncology, Bangalore, Karnataka, India; 3 Department of Head and Neck Oncology, Kidwai Memorial Institute of Oncology, Bangalore, Karnataka, India

**Keywords:** lymphoplasmacytic, nasopharynx, Waldenstrom’s macroglobulinemia, COP

## Abstract

We report a rare case of a 68-year-old male who presented with fever, weight loss, nasal blockage, and epistaxis. Examination revealed cervical and axillary lymphadenopathy with no evidence of organomegaly. On evaluation, bone marrow aspiration showed lymphoplasmacytic infiltration. The computed tomography of the neck showed nasopharyngeal mass and the biopsy of this mass and cervical lymph node showed lymphoplasmacytic lymphoma (LPL) with high serum IgM level. Hence, a diagnosis of Waldenstrom’s macroglobulinemia (WM) was made. The patient received six cycles of chemotherapy with a combination of cyclophosphamide, vincristine, and prednisolone (COP regimen). Currently, the patient is under follow-up and in complete remission (CR), one year after completion of therapy. Nasopharyngeal involvement is extremely rare in WM, and hence we report this case.

## Case report

**Figure 1.**
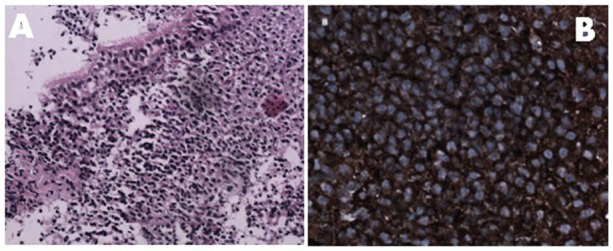
Panel A: Monotonous appearance of lymphocytes and plasmacytoid lymphocytes beneath the nasopharyngeal epithelium [H & E 20 X]. Panel B: Tumour cells expressing membranous kappa light chain positivity [40 X].

**Figure 2.**
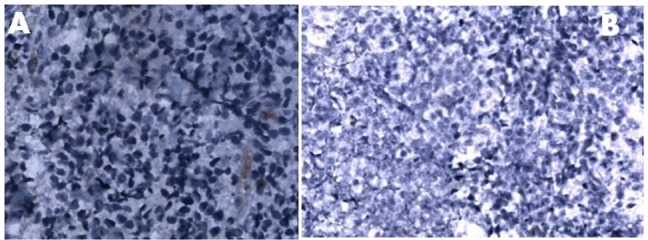
Panel A: Absence of expression of lambda light chain [40 X]. Panel B: Immunohistochemistry (IHC) showing cyclin D1 negativity [20 X].

A 68-year-old gentleman presented with bilateral neck swelling, intermittent fever, and fatigue of one-month duration. He also had a history of weight loss of 4 kg in one month. He had a history of intermittent nasal blockage and one episode of epistaxis. He denied a history of pruritus, chest pain, abdominal pain, and urinary or bowel disturbances. He also had a history of smoking one pack of cigarettes for the last 40 years. He was a known hypertensive on treatment with antihypertensive medications. On examination, he had an Eastern Cooperative Oncology Group performance status of one. There was no evidence of pallor or icterus. Enlarged bilateral cervical levels I B, II, III, IV, and V multiple discrete lymph nodes, the largest measuring 3 × 2 cm^2^ on the right and 2 × 1 cm^2^ on the left, were palpable. A bilateral central group of axillary lymph nodes, the largest measuring 3 × 3 cm^2^, was evident. Examination of the heart, abdomen, and lungs was unremarkable. On evaluation, his haemogram showed a haemoglobin of 11 g%, white count of 8,600/cumm, and thrombocyte count of 198,000/cumm. His random blood sugar, renal parameters, liver function tests, and serum lactate dehydrogenase levels were within normal limits. Serum for human immune deficiency virus and hepatitis B and C markers were negative. Electrocardiogram and echocardiogram were normal. The computed tomography (CT) of the neck revealed multiple homogeneously enhancing lymph nodes in bilateral levels II, III, IV, and V, the largest 3 × 3.5 cm^2^ on the right and 3 × 2.8 cm^2^ on the left. His nasopharynx appeared bulky and there was a mass lesion measuring 3.2 × 3.4 cm^2^. The CT of the chest and abdomen was unremarkable apart from bilateral axillary lymphadenopathy. Cervical lymph node and nasopharyngeal mass biopsies (Figures 1 and 2) were done, which showed neoplastic cells positive for CD 20, bcl 2, CD 5, and kappa and negative for CD 10, CD 23, cyclin D1, CD3, and lambda. Ki 67 index was 15% suggestive of low-grade B cell lymphoma with plasmacytic differentiation—LPL. Serum beta 2 microglobulin was elevated (4.5 mg/L), serum protein electrophoresis had M spike in gamma region of 0.89 gm/dL. A blood test for free light chain assay was done, and it showed an increased free kappa (291 mg/L) and elevated kappa/lambda ratio of 15.9. Serum immunoglobulin M levels were high (13.73 g/L). The skeletal survey (skull, vertebrae, chest, and hip) did not reveal any osteolytic lesions. Bone marrow aspiration showed marrow involvement by lymphoma with lymphoplasmacytic infiltration (lymphocytes of 60% with plasma cells of 10%). Bone marrow cytogenetics did not yield any metaphases. Although we advised the patient for bone marrow fluorescent *in situ *hybridisation (FISH) assay, it could not be done due to financial constraints. Correlating with clinical, increased serum IgM levels, bone marrow findings, and histopathological findings, a diagnosis of WM was made. The patient was counselled regarding the nature of his illness, and he received six cycles of chemotherapy 3q weekly with a combination of cyclophosphamide (750 mg/m^2^), vincristine (1.4 mg/m^2^), and prednisolone (100 mg/day for five days). Currently, the patient is under follow-up and in CR one year after completion of therapy.

## Discussion

Various classifications have evolved over the last two decades to diagnose this low-grade lymphoma (lymphoplasmacytic) including the Rappaport (well-differentiated lymphocytic and plasmacytoid), Lukes–Collins (plasmacytic–lymphocytic), Kiel (lymphoplasmacytic), working formulation (small lymphocytic and plasmacytoid), revised European American Lymphoma (LPL), and the World Health Organisation (WHO; WM) [1].

WM was named after the Swedish oncologist Jan G. Waldenström in 1944 who reported two patients with epistaxis, hypofibrinogenemia, lymphadenopathy, neoplastic plasma cells in bone marrow, and macroglobulinemia [2].

The 2008 WHO classification defined WM as a mature B-cell lymphoid neoplasm composed of small B lymphocytes, plasmacytoid lymphocytes, and plasma cells, usually involving bone marrow and sometimes lymph nodes and, very rarely, the spleen, which does not fulfil the criteria for any of the other small B-cell lymphoid neoplasms [3]. It is a diagnosis of exclusion and when associated with IgM monoclonal gammopathy, it is termed as WM [4].

The overall incidence of WM is approximately five cases per one million persons per year. The incidence has remained steady over time as suggested by Wang *et al *[5], who reported an incidence of 0.38 per 100,000 persons per year. It accounts for ~1–2% of haematologic malignancies. The incidence is highest among white people and is rare in other population groups. The median age at diagnosis varies between 63 and 68 years, and has a preponderance for men [5]. This entity is not very common in India, and our patient was a 68-year-old male who presented with generalised lymphadenopathy and epistaxis.

The aetiology is unclear, and no specific environmental or occupational exposure including smoking has been linked to this entity. In most of the cases, it appears to be sporadic; however, there have been reports of familial clustering.

Possible associations between the hepatitis C virus (HCV) and human herpes virus-8 and WM have been suggested, but the association of HCV with WM was refuted in [6]. Our patient was negative for HCV ELISA.

The incidence of LPL presenting with B symptoms is 23% in western studies as compared with 78% in a study done by Sajid *et al *in 2010 in Pakistan [7]. In our case, the patient had B symptoms, which characteristically involved the bone marrow and lymph nodes. Extra nodal involvement and leukaemic phase is quite rare. Our patient had intermittent nasal blockage and lymphadenopathy with no evidence of organomegaly. Features related to monoclonal gammopathy, including hyperviscosity, cryoglobulinemia, and amyloidosis, may be observed. LPL/WM can involve various tissues such as the skin, gastrointestinal tract, kidney, liver, adnexa, minor salivary gland, central nervous system, and retina. Involvement of the nasopharynx is extremely rare, and only one patient with WM associated with extranodal marginal zone B cell–mucosa-associated lymphoid tissue lymphoma has been previously reported [8]. The present case is probably the first of its kind to the best of our knowledge, as we conducted a literature search with isolated WM having nasopharyngeal involvement.

Bone marrow morphology shows a monotypic lymphocytic component in LPL, typically with high levels of surface CD19, CD20, and immunoglobulin light chain expression. The lymphoid cells lack CD10. In 30% of cases, the lymphocytes show some degree of CD5 expression, which can be confused with chronic lymphocytic leukaemia (CLL)/small lymphocytic lymphoma or mantle cell lymphoma as also observed in our case.

The plasmacytic component expresses the same immunoglobulin light chain as the lymphocytic component is positive for CD138 and shows diminished expression of B-cell-associated antigens such as CD19, CD20, and PAX5. The LPL cells are positive for surface IgM. WM cells have also been shown to be CD25^+^, CD27^+^, CD75^–^, FMC7^+^, Bcl2^+^, and Bcl6^–^. Del(6)(q21) is the most common genetic abnormality observed in 40–50% of cases, and this genetic abnormality is rarely observed in other lymphoproliferative or plasmaproliferative malignancies [8, 10]. Cervical lymph node and nasopharyngeal mass biopsies were done, which showed neoplastic cells positive for CD 20, bcl 2, CD 5, and kappa and negative for CD 10, CD 23, cyclin D1, CD3, and lambda. Ki 67 index was 15% suggestive of low-grade B cell lymphoma with plasmacytic differentiation. Correlating with high serum IgM levels, we made a diagnosis of WM.

WM is a diagnosis of exclusion and other entities should also be considered. Clonal B lymphoplasmacytic infiltration in bone marrow with increased IgM levels can be observed in other conditions like splenic marginal zone lymphoma (SMZL). SMZL only rarely presents with lymphadenopathy and always has splenomegaly in contrast to the presentation with lymphadenopathy in our patient. However, in our patient, there was no evidence of splenomegaly and circulating villous lymphocytes, which is the characteristic of splenic lymphoma with villous lymphocytes. In addition, the *k* / *l *ratio is 1.2:1 for SMZL and 4.5:1 for WM, which could also be a differentiating point. In our patient, the *k* / *l* ratio was 15.9 going in favour of WM [4, 11].

Loss of 7q along with +3q and +5q and CD22 and CD11c overexpression are observed in SMZL. Nodal marginal zone lymphoma could also be a differential diagnosis for WM. Both NMZL and WM present with lymphadenopathy; however, bone marrow involvement is observed in only one-third of patients with NMZL, while it is more common in WM. Moreover, CD5 and CD23 are usually negative, being reported in less than 10% of patients with NMZL. In our patient CD 5 was positive. These factors helped us to rule out NMZL [4, 11]. 

In patients without symptoms, monoclonal IgM elevation and with bone marrow plasma cells less than 10%, IgM-monoclonal gammopathy of unknown significance (MGUS) should also be kept in the list of differential diagnosis. However, in our case, the patient was symptomatic and bone marrow plasma cells were 10%. FISH is highly useful in differentiating WM from IgM-MGUS as (6q-) is not observed in IgM MGUS and is observed in WM. Due to financial constraints, our patient could not undergo a FISH [12].

B-cell CLL may mimic WM, as also observed in our case, as both can present with lymphadenopathy. However, bone marrow morphology and immunophenotyping can help to differentiate the two. Our patient had CD 20, bcl 2, and CD 5 positivity and CD23 negativity in contrast to B-CLL, which would have both CD 5 and 23 positivity [3]. The differential diagnosis of WM and the criteria to differentiate it from other entities have been summarised in Table 1.

A ‘watch-and-wait’ approach can be adopted in patients with WM who are asymptomatic, with nonbulky disease, and no signs and symptoms of organ failure. Various drugs, including chlorambucil, melphalan, cyclophosphamide, cladribine, fludarabine, rituximab, bortezomib, ofatumumab, alemtuzumab, and thalidomide, have been tried. A combination regimen that includes alkylating agents, vinca alkaloids, and anthracyclines has been used with reasonable results. Rituximab in combination with alkylating agent is the preferred initial therapy for WM who have B symptoms, haematologic compromise, bulky disease, or symptoms of hyperviscosity. It is mandatory to carry out plasmapheresis before starting chemotherapy for those patients who present with symptoms of hyperviscosity [13, 14]. We used a combination of cyclophosphamide, vincristine, and prednisolone (COP) in our patient. Rituximab could not be used in our patient as he had financial constraints. The patient maintained CR after six cycles of chemotherapy and has been under follow-up for the last one year with CR.

## Conclusion

WM with nasopharyngeal involvement is extremely rare; hence, we report this case of clinical importance. Clinicians should consider this diagnosis along with other low-grade B cell lymphomas. Although nasopharyngeal involvement is common in other lymphomas like mantle cell lymphoma, WM should be kept in the differential diagnosis as observed in our case.

## Figures and Tables

**Table 1.** Differential diagnosis of WM.

Disease	Criteria to differentiate from WM
**Splenic marginal zone lymphoma**	Splenomegaly is more common Higher CD22 and CD11c Less CD25 and CD103 is positive in SMZL but negative in WM SMZL is characterised by loss of 7q (19%) along with +3q (19%) and +5q (10%), whereas WM is characterised by 6q deletion MYD88 mutation occurs in the majority of patients with WM but in only 10% of SMZL patients
**Nodal marginal zone lymphoma**	Marrow involvement is less common and CD5 and CD23 negative
**Extra nodal marginal zone lymphoma**	Pure monocytoid morphology is more typical of MALT lymphoma than NMZL, specific translocation involving BCL10 and MALT1
**IgM MGUS**	<10% LPL cells in the BM and <3 g of Ig with no symptoms related to WM
**IgM MM**	Plasma cells in the BM, cytogenetics and FISH studies identify MM-related abnormalities, specifically t(11;14) Presence of lytic lesions Absence of MYD88 mutation
**Mantle cell lymphoma**	Involves lymph nodes and extranodal sites t(11;14) (q13;q32) almost in all cases
**B-CLL**	CD5 and CD23 positive, CD10 negative
**Follicular lymphoma**	Small cleaved lymphocytes with Bcl-2 re-arrangement

WM, Waldenstrom’s macroglobulinaemia; MALT, mucosa-associated lymphoid tissue lymphoma.
